# Supplementary material for: Glutacetine® Biostimulant Applied on Wheat under Contrasting Field Conditions Improves Grain Number Leading to Better Yield, Upgrades N-Related Traits and Changes Grain Ionome
Source: Plants (Basel). 2021 Feb 28;10(3):456. doi: 10.3390/plants10030456 (PMC7997451; doi:10.3390/plants10030456)
Supplement: Supplementary file 1 [file plants-10-00456-s001.pdf]

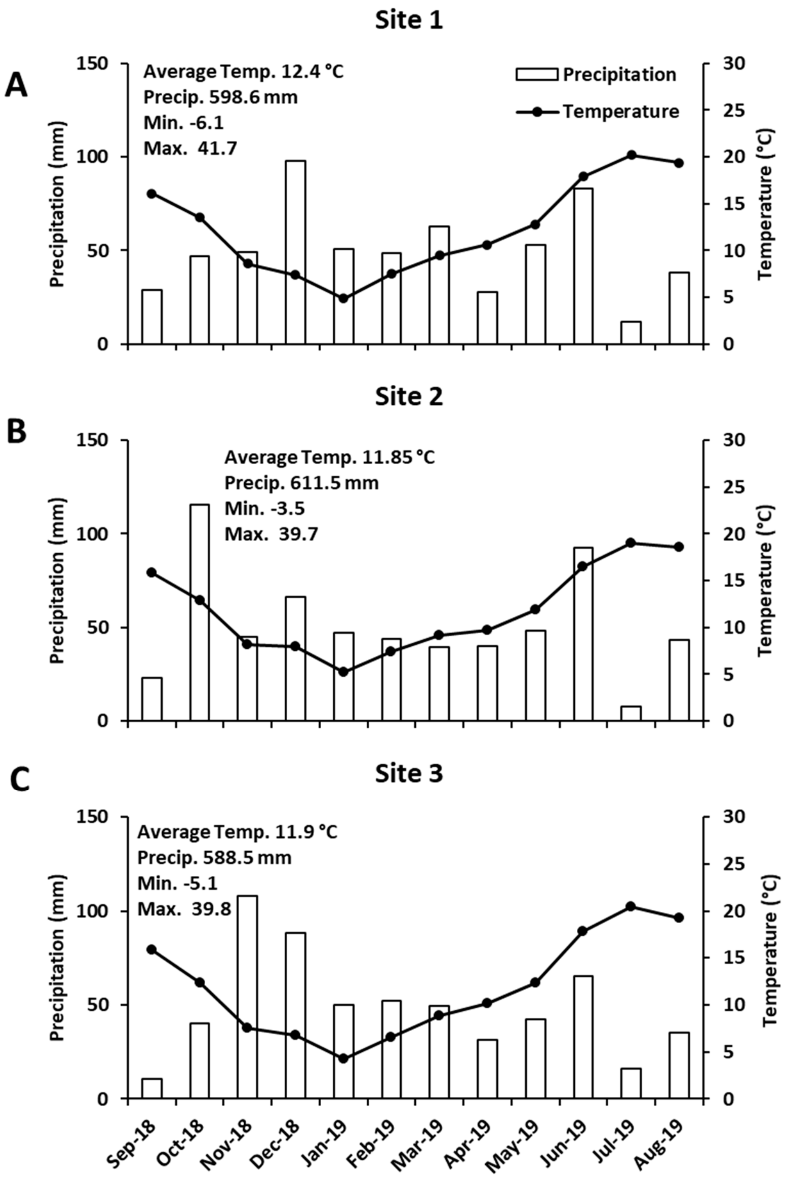

Supplementary Figure S1. Weather conditions for each site during the growing period. (A) Site 1, (B) Site 2 and (C) Site 3.

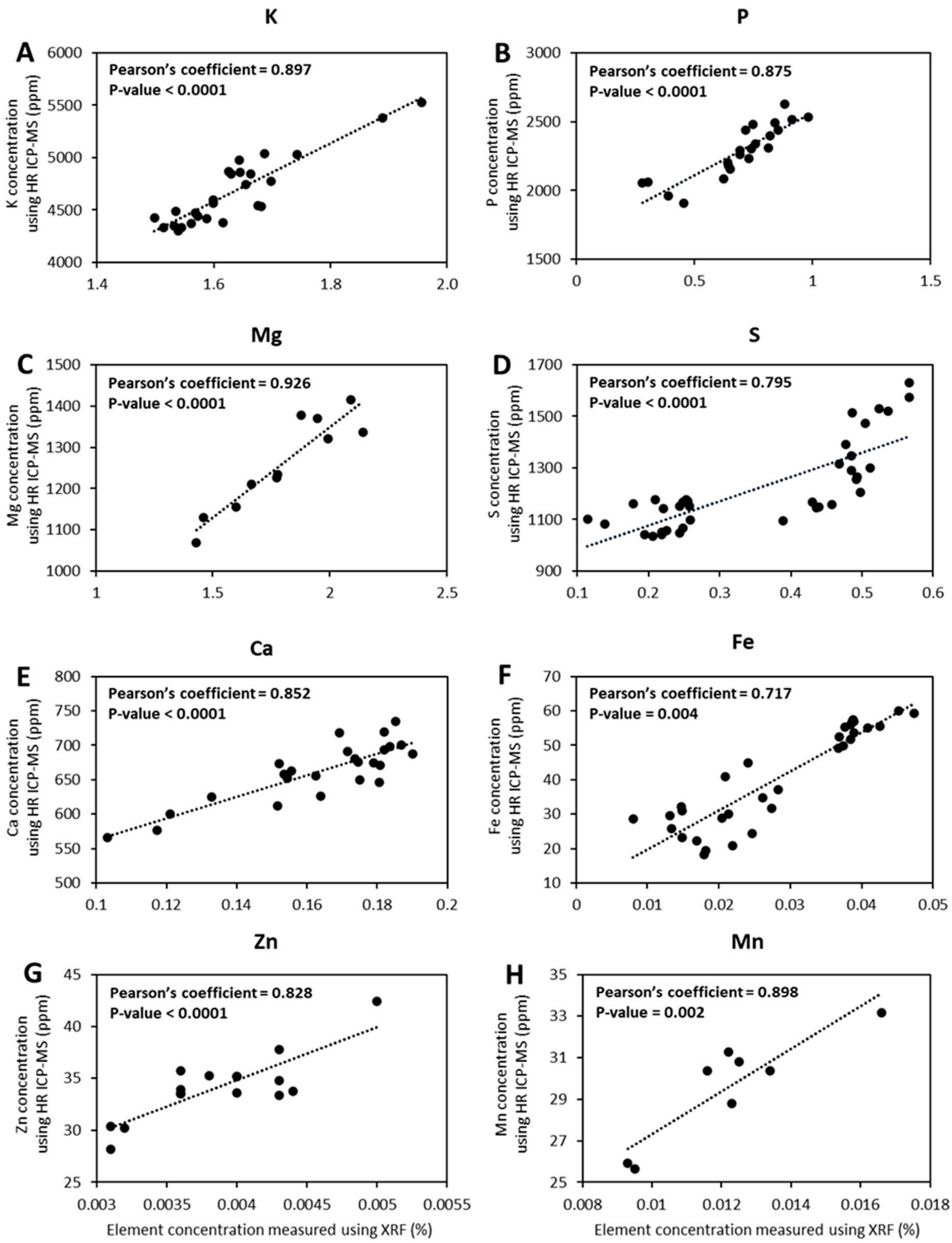

Supplementary Figure S2. Relationship between element concentrations of wheat flour (*Triticum aestivum* L.) values obtained with the HR ICP-MS and XRF methods. (A) K content, (B) P content, (C) Mg content, (D) S content, (E) Ca content, (F) Fe content, (G) Zn content and (H) Mn content.

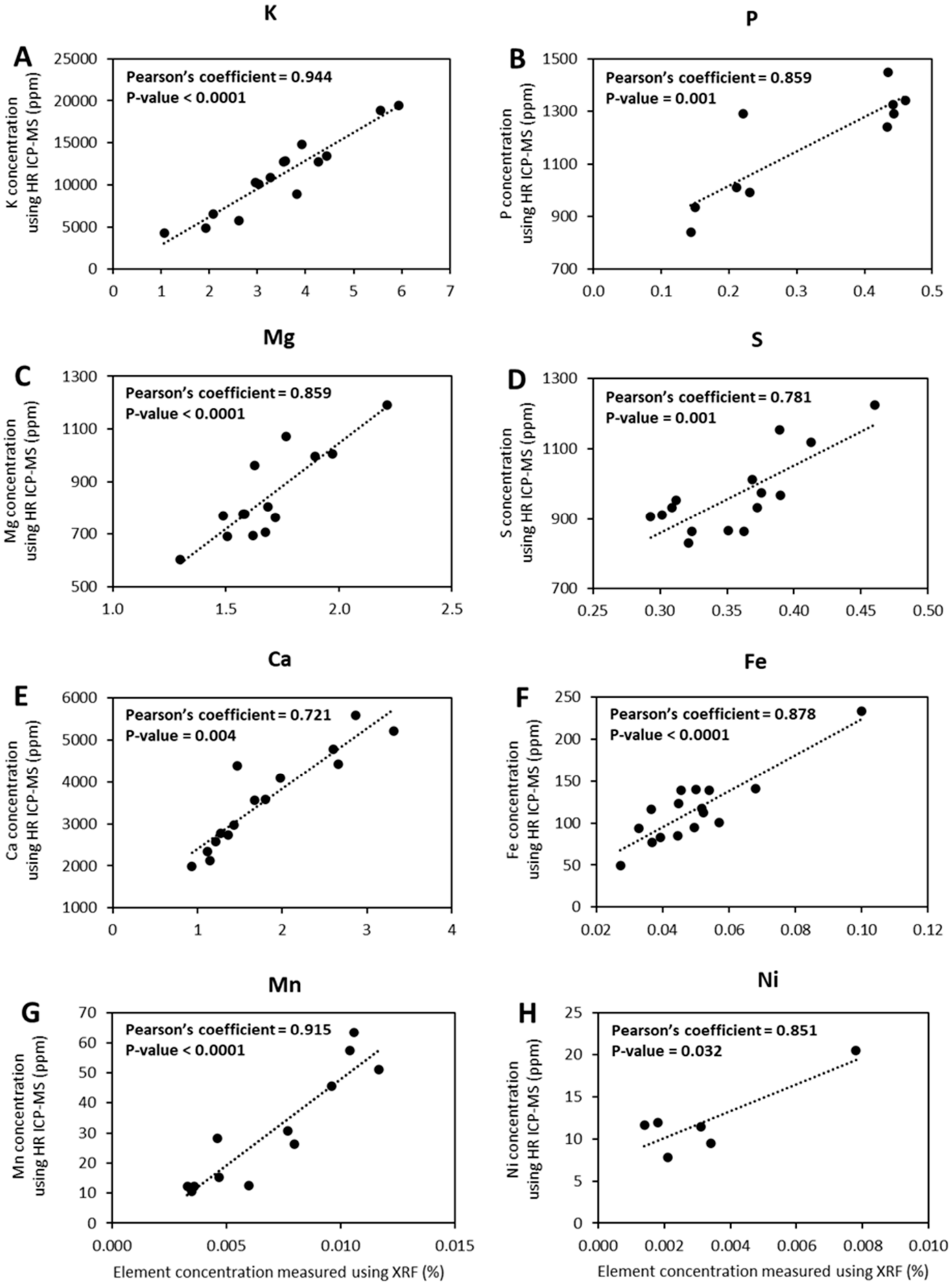

Supplementary Figure S3. Relationship between element concentrations of wheat straw (*Triticum aestivum* L.) values obtained with the HR ICP-MS and XRF methods. (A) K content, (B) P content, (C) Mg content, (D) S content, (E) Ca content, (F) Fe content, (G) Mn content and (H) Ni content.

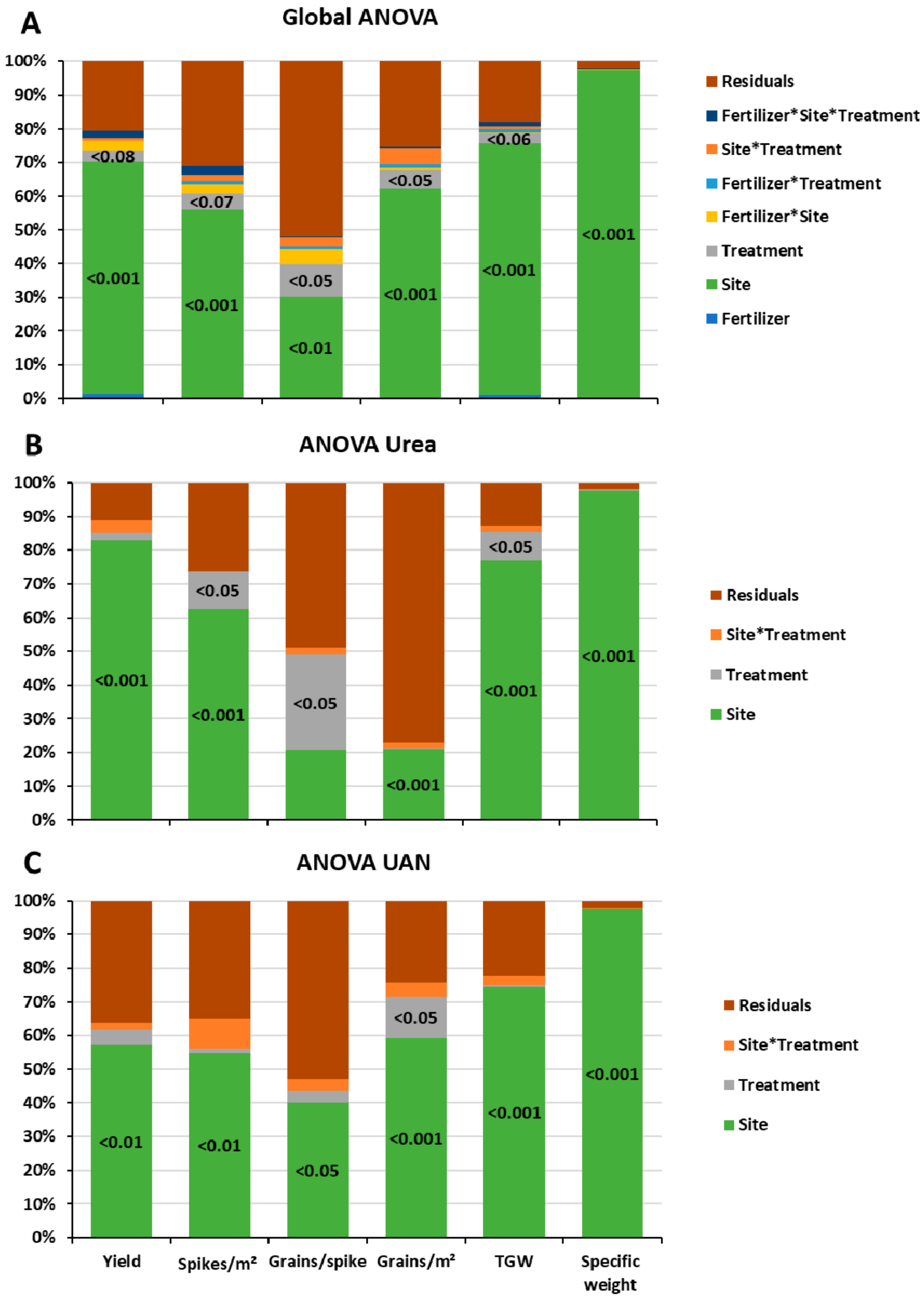

Supplementary Figure S4. Schematic representation of the ANOVA for yield, spike number per square metre, grain number per spike, grain number per square metre, thousand grain weight (TGW) and specific weight. (A) Global ANOVA using data obtained in the three sites and with the two fertilizers, (B) ANOVA Urea using data obtained from the three sites with urea fertilizer and (C) ANOVA UAN using data obtained from the three sites with UAN fertilizer.

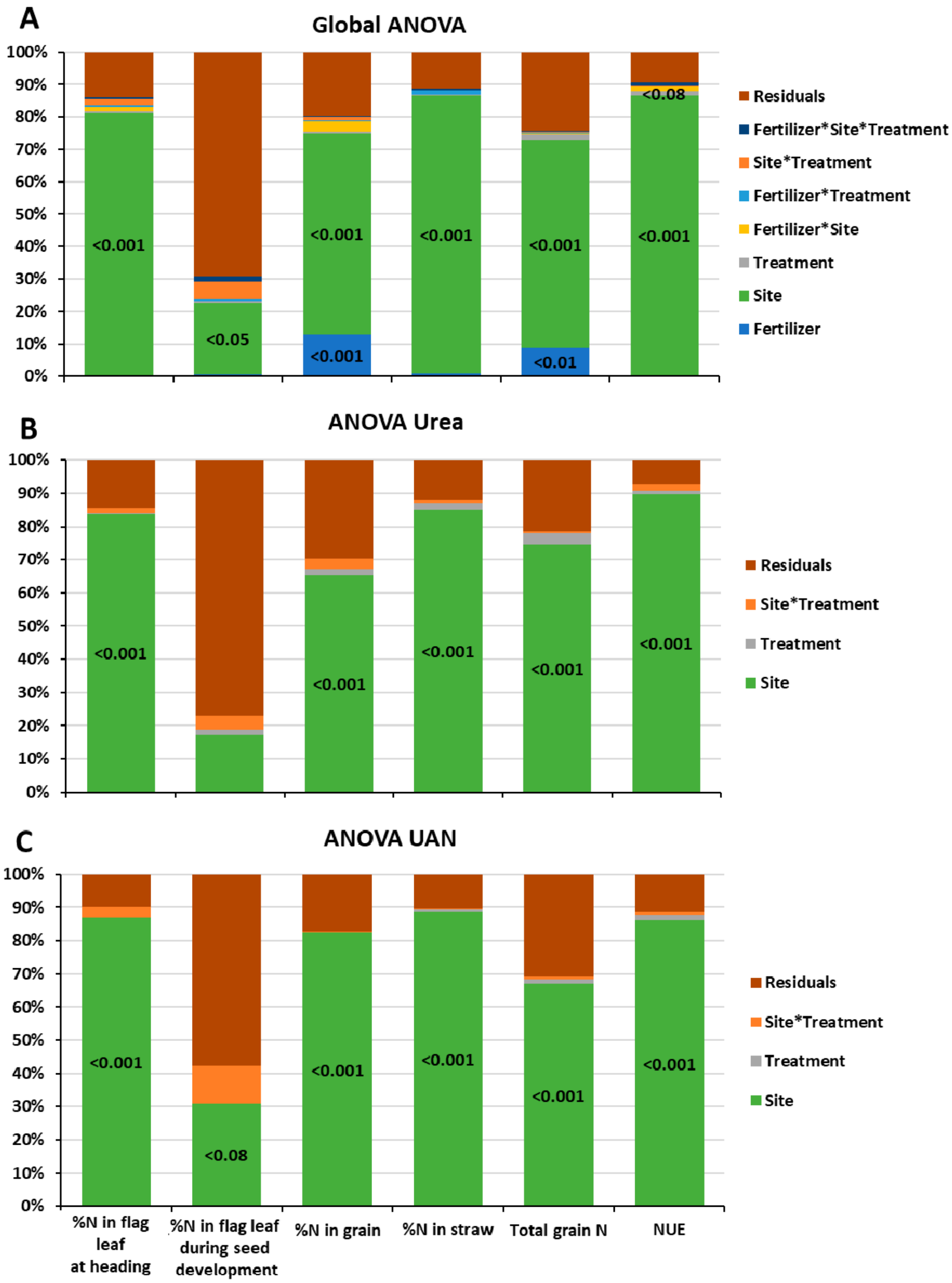

Supplementary Figure S5. Schematic representation of the ANOVA of the %N in flag leaf at heading, %N in flag leaf during seed development, %N in grain, %N in straw, total grain N and N use efficiency (NUE). (A) Global ANOVA using data obtained in the three sites and with the two fertilizers, (B) ANOVA Urea using data obtained from the three sites with urea fertilizer and (C) ANOVA UAN using data obtained from the three sites with UAN fertilizer.

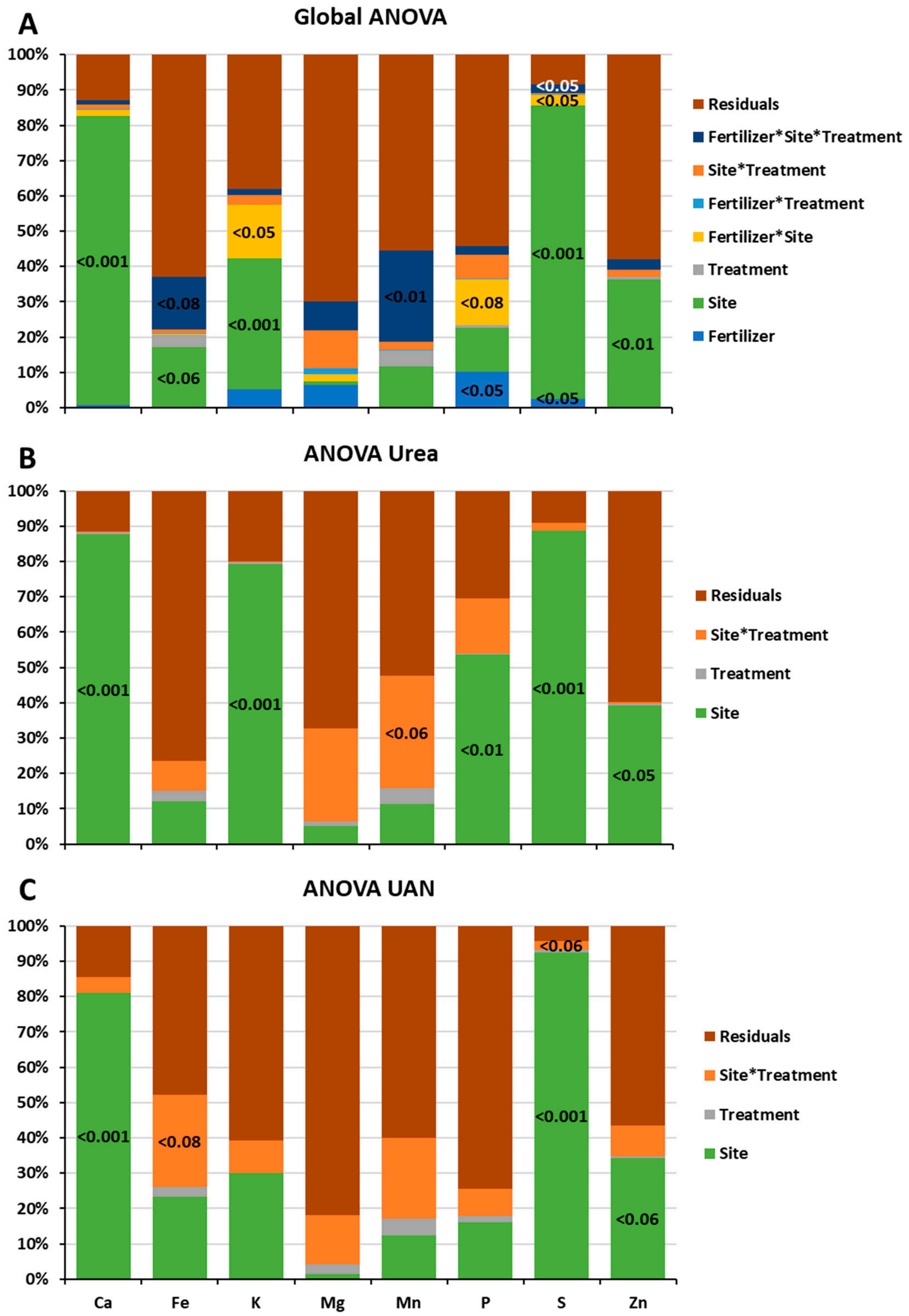

Supplementary Figure S6. Schematic representation of the ANOVA of the grain ionome (elements detected in all samples by XRF; Ca, Fe, K, Mg, Mn, P, S and Zn). (A) Global ANOVA using data obtained in the three sites and with the two fertilizers, (B) ANOVA Urea using data obtained from the three sites with urea fertilizer and (C) ANOVA UAN using data obtained from the three sites with UAN fertilizer.

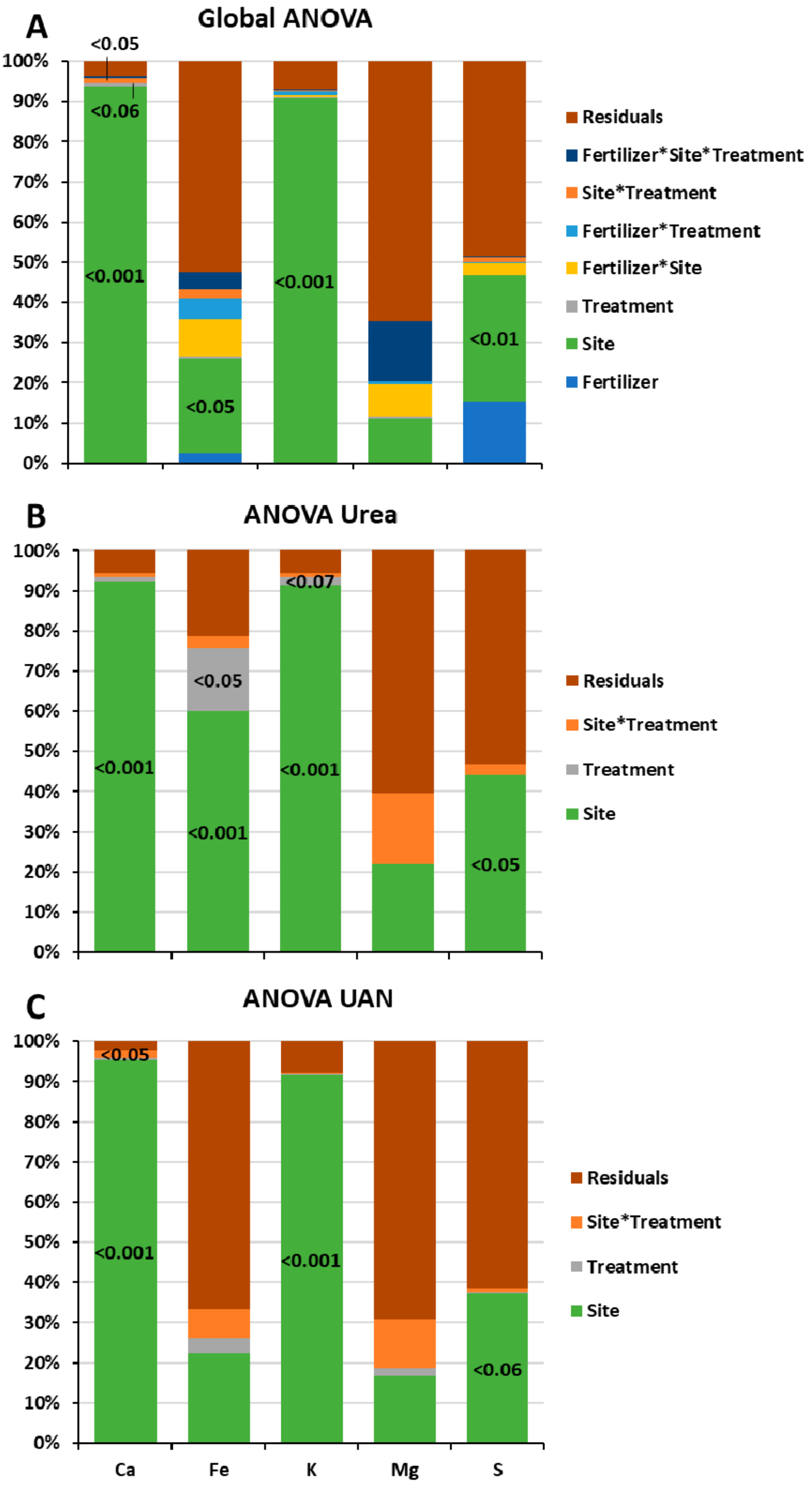

Supplementary Figure S7. Schematic representation of the ANOVA of the straw ionome (elements detected in all samples by XRF; Ca, Fe, K, Mg and S). (A) Global ANOVA using data obtained from the three sites and with the two fertilizers, (B) ANOVA Urea using data obtained from the three sites with urea fertilizer and (C) ANOVA UAN using data obtained from the three sites with UAN fertilizer.

Supplementary Table S1. Location, wheat cultivars and soil properties of the three field trials

	Site 1	Site 2	Site 3
**Location**	49.26491, -0.87763	49.512660, 0.409829	48.851115, 0.023732
**Previous crop**	*Beta vulgaris*	*Linum usitatissimum*	*Medicago sativa*
**Wheat cultivar**	Sacramento	Libravo	Chevignon
**Sowing date**	17-11-2018	16/10/2018	16-10-2018
**Organic matter input**	Yes	No	No
**Soil type**	Clay loams / Frank loams	Deep silt	Superficial clay limestone
**pH (water)**	6.2	7.3	8.4
**CEC (mol kg^{-1})**	9.5	8.5	10.8
**Organic matter (%)**	2.4	2.1	3.4
**C/N Ratio**	Not determined	8.1	7.6
**Total N (%)**	Not determined	0.15	0.26
**Organic C (%)**	1.42	1.22	1.98
**Available P (ppm)**	132	101	62
**Exchangeable K (ppm)**	168	238	254
**Exchangeable Mg (ppm)**	104	99	216
**CaO (‰)**	2.1	3.6	12.3

Supplementary Table S2. Nitrogen management in the three field trials

	**Site 1**	**Site 2**	**Site 3**
**Humus effect**	65	50	30
**Previous crop effect**	20	0	40
**M.O. supply effect**	12	0	0
**N uptake**	10	29	26
**N residue (NO_3^-/NH_4^+)**	33 (25/8)	35 (33/2)	42 (34/8)
**Total N soil supply (kgN ha^{-1})**	140	114	138
**Crop needs**	272	304	270
**N remaining**	20	20	20
**Total N needs**	292	324	290
**N fertilizer**	152	210	152
**Supply 1 (Tillering, BBCH 21)**	61	100	62
**Supply 2 (Stem elongation, BBCH 31)**	31	50	30
**Supply 3 (Flag leaf extended), BBCH 39)**	60	60	60
